# Navigating the Challenges of Deep Inferior Epigastric Artery Perforator (DIEAP) Flap Harvest in a Scarred Abdomen

**DOI:** 10.7759/cureus.103142

**Published:** 2026-02-07

**Authors:** Smruti Srinivasan, Neeraj Rao, Akshay Kapoor, Farhanul Huda

**Affiliations:** 1 Plastic and Reconstructive Surgery, All India Institute of Medical Sciences, Rishikesh, IND; 2 General Surgery, All India Institute of Medical Sciences, Rishikesh, IND

**Keywords:** abdominal scars, breast q score, breast reconstruction, diep flap, intraoperative challenges, perforator dissection, venous supercharging

## Abstract

Background

The deep inferior epigastric artery perforator (DIEAP) flap is the gold standard for autologous breast reconstruction, offering reliable perfusion and minimal donor-site morbidity. However, prior abdominal surgeries and scars may complicate flap harvest by altering vascular pathways, causing fibrosis, and limiting flap design. This study evaluates intraoperative challenges, outcomes, and patient-reported satisfaction in DIEP flap reconstruction in scarred abdomens.

Methods

A prospective observational study was conducted at a tertiary care plastic surgery center (2022-2024). A total of 20 female patients with visible abdominal scars undergoing immediate or delayed DIEAP flap reconstruction were included. All underwent CT angiography (CTA) for perforator mapping. Data collected included demographics, scar types, intraoperative findings, complications, and postoperative outcomes. The BREAST-Q reconstruction module scores were used to assess patient satisfaction.

Results

The mean age was 40.6 years (SD 8.9) with a mean hospital stay of 9.2 days (SD 2.9). Indications included invasive breast carcinoma (n=16) and phyllodes tumor (n=4). Scar types included Pfannenstiel (n=14), subcostal (n=6), periumbilical (n=3), and paramedian (n=2); seven patients had multiple scars. Intraoperative challenges included fibrosis, difficult perforator dissection, and restricted flap design. One index case with Pfannenstiel and periumbilical scars demonstrated superficial venous dominance requiring venous supercharging, which salvaged the flap. Recipient-site complications occurred in two patients (10%), and donor-site complications in four (20%). All flaps survived, with no total flap loss. The BREAST-Q scores demonstrated high psychosocial satisfaction (mean 81.5) and strong surgeon-related outcomes, though abdominal satisfaction was lower (64.8).

Conclusions

The DIEAP flap reconstruction in scarred abdomens is safe and effective, though scar pattern significantly influences planning and harvest. Preoperative CTA and intraoperative adaptability, including additional procedures like venous supercharging when necessary, enable excellent outcomes even in complex cases.

## Introduction

The deep inferior epigastric artery perforator (DIEAP) flap has become the gold standard for autologous whole breast reconstruction, offering reliable vascularity, excellent aesthetic outcomes, and minimal donor-site morbidity by preserving the rectus abdominis muscle [1]. However, a growing number of breast reconstruction recipients present with prior abdominal surgeries, most commonly caesarean sections, cholecystectomies, appendicectomies, laparoscopic procedures, and laparotomies, which pose unique challenges during flap planning and harvest.

The presence of abdominal scars may distort normal anatomy, compromise the course or calibre of perforators, or result in dense subcutaneous and intramuscular fibrosis. These changes increase the technical complexity of flap dissection, elevate the risk of perfusion-related complications, and may necessitate intraoperative modifications [2,3]. While several studies have addressed the feasibility of DIEAP flaps in scarred abdomens [4-6], there remains limited literature specifically detailing outcomes in patients with multiple or complex abdominal surgical histories.

This study is unique in that it focuses specifically on the intraoperative challenges posed by prior abdominal scars in DIEAP flap breast reconstruction, based on a prospective analysis conducted at a tertiary care plastic surgery centre. The study underscores how the pattern, location, and number of abdominal scars can influence perforator selection, surgical planning, and intraoperative decision-making. Special emphasis is placed on the index patient who had undergone two lower-segment caesarean sections (LSCS) and a laparoscopic myomectomy, resulting in dense periumbilical scarring, which required additional procedures for salvage of the flap.

The primary objective of this prospective observational study was to assess the impact of pre-existing abdominal scars on intraoperative challenges, flap design constraints and surgical decision-making during DIEAP flap breast reconstruction, and document the need for intraoperative modifications such as altered flap design or venous supercharging wherever required. The secondary objectives were to assess donor-site and recipient-site outcomes and evaluate patient-reported outcomes following reconstruction in this cohort.

## Materials and methods

Study design

This is a prospective, single-centre, observational study evaluating the intraoperative challenges and outcomes of DIEAP flap breast reconstruction in patients with pre-existing abdominal scars. The study specifically documented the influence of scar pattern, location, and number on surgical planning, flap selection, intraoperative modifications, and postoperative outcomes. Intraoperative challenges were defined as difficulty in perforator dissection due to dense fibrosis or altered anatomy, limited flexibility in flap design imposed by scar location, compromised venous outflow requiring intraoperative modification, or the need for altered perforator or flap selection. A total of 20 female patients who underwent immediate or delayed DIEAP flap breast reconstruction were included. All participants had a documented history of previous abdominal surgery resulting in visible abdominal scars.

This prospective observational study was conducted at a tertiary care plastic surgery centre. The study protocol was approved by the Institutional Ethics Committee (IEC) of the All India Institute of Medical Sciences (Rishikesh, UK, IND) and adhered to the principles of the Declaration of Helsinki and local institutional regulations. Written informed consent for participation, data use, and inclusion of anonymised images was obtained from all patients. All patients were followed up for a minimum of six months postoperatively to assess both surgical and patient-reported outcomes.

Study measures

Patient demographics, history of prior abdominal surgeries, type and location of scars and data of preoperative CT angiography (CTA) were included. The CTA was performed using a standard arterial-phase abdominal protocol. The CTA images were reviewed for the number, calibre, and intramuscular course of perforators, their relationship to existing scars, and the presence or dominance of the superficial venous system. These findings guided preoperative perforator selection and flap design.

Perforator selection was based on calibre, pulsatility, intramuscular course, and location relative to scarred tissue, with preference given to perforators outside dense fibrotic zones. Flap configuration (unipedicle, bipedicle, or bilateral unipedicle) was individualised based on perforator distribution, scar burden, and reconstructive requirements.

The internal mammary vessels were used as the primary recipient vessels in all cases. In situations of suspected venous congestion or dominant superficial venous drainage, venous supercharging was performed, typically by anastomosing the superficial inferior epigastric vein to the cephalic vein.

Intraoperative findings, flap types (unipedicle/bipedicle), modifications in the flap design or the surgical procedure and challenges faced were also documented. Postoperative parameters included recipient and donor-site complications, duration of hospital stay and patient-reported outcomes assessed using the BREAST-Q reconstruction module, which was administered at a minimum postoperative follow-up of six months. The questionnaire was completed either during routine outpatient follow-up visits or through structured interviews conducted by the treating team. Scores were calculated according to the standardised BREAST-Q scoring manual, with higher scores indicating greater satisfaction or well-being.

Statistical analysis

All data were compiled and analysed using Microsoft Excel (Microsoft Corp., Redmond, WA, USA) and SPSS Statistics version 26.0 (IBM Corp., Armonk, NY, USA). Descriptive statistics were used to summarise demographic and clinical data. Continuous variables were expressed as mean ± SD, and categorical data as frequency and percentage. No inferential statistics were applied due to the limited sample size and descriptive nature of the study. The BREAST-Q scores across domains were presented as mean ± SD values to provide a comparative overview of patient satisfaction. Given the descriptive and exploratory nature of this study, the heterogeneity of scar patterns, and the limited sample size, no inferential statistical analyses were performed, and results are presented using descriptive statistics only.

## Results

The mean age of the patients was 40.6 years (SD: 8.9), ranging from 27 to 56 years. The most common indications for reconstruction were invasive breast carcinoma (n=16), followed by a phyllodes tumour (n=4). The mean duration of hospital stay was 9.2 days (SD: 2.9), with the shortest being six days and the longest 16 days.

Fourteen patients (70%) had undergone one or more LSCS, all resulting in Pfannenstiel scars. Six patients (35%) had undergone open cholecystectomy, resulting in right subcostal scars. Three patients had an infraumbilical port scar from laparoscopic surgeries. Two patients had a lower paramedian scar. Seven out of 20 patients had multiple abdominal scars, including combinations of Pfannenstiel and subcostal, Pfannenstiel and periumbilical, and Pfannenstiel and drain-site scars (Table 1).

**Table 1:** Summary of patient characteristics, abdominal scar patterns, and postoperative outcomes in DIEAP flap breast reconstruction DIEAP: Deep inferior epigastric artery perforator, ALND: Axillary lymph node dissection, MRM: Modified radical mastectomy, NSM: Nipple sparing mastectomy, SSM: Skin-sparing mastectomy, LSCS: Lower-segment cesarean section, NAC: Nipple areolar complex

Patient no.	Age (years)	Diagnosis	Type of mastectomy	Timing of reconstruction	Type of flap	Previous abdominal surgery	Site of abdominal scar	Length of hospital stay (days)	Recipient site complications	Donor site complications
1	53	Invasive breast carcinoma	Nipple sparing mastectomy + axillary lymph node dissection (ALND)	Immediate	Bipedicle	Open cholecystectomy	Subcostal scar	7	None	None
2	33	Invasive breast carcinoma	Modified radical mastectomy (MRM)	Immediate	Unipedicle	2 LSCS + Open cholecystectomy	Pfannenstiel + Subcostal scar	7	None	None
3	41	Invasive breast carcinoma	MRM	Immediate	Unipedicle	Classic cesarean section	Lower paramedian scar	8	None	None
4	34	Recurrent phylloides tumor	Nipple sparing mastectomy (NSM)	Immediate	Unipedicle	2 LSCS	Pfannenstiel scar	7	Partial nipple areolar complex (NAC) necrosis	None
5	48	Bilateral invasive ductal carcinoma	Right MRM, Left NSM + ALND	Immediate	Bilateral unipedicle	1 LSCS + Lap myomectomy	Pfannenstiel + periumbilical	15	None	Abdominal hematoma
6	28	Malignant phylloides tumor	Simple mastectomy	Immediate	Unipedicle	2 LSCS	Pfannenstiel scar	6	None	None
7	38	Invasive breast carcinoma	MRM	Immediate	Unipedicle	2 LSCS + Fallopian tube surgery	Pfannenstiel + drain scar	13	None	Abdominal wound dehiscence
8	38	Invasive breast carcinoma	MRM	Immediate	Unipedicle	Open cholecystectomy	Subcostal scar	8	None	None
9	–	Invasive breast carcinoma	NSM	Immediate	Unipedicle	2 LSCS + Lap cholecystectomy	Pfannenstiel + periumbilical	8	None	None
10	48	Invasive breast carcinoma + BRCA+	Right skin-sparing mastectomy (SSM) + left prophylactic SSM	Immediate	Bilateral unipedicle	2 LSCS	Pfannenstiel scar	10	None	None
11	32	Invasive breast carcinoma	MRM	Immediate	Unipedicle	LSCS ×1	Pfannenstiel scar	8	None	None
12	45	Invasive breast carcinoma	MRM	Immediate	Unipedicle	Open cholecystectomy	Subcostal scar	9	None	Abdominal wound dehiscence
13	36	Malignant phyllodes tumor	Simple mastectomy	Immediate	Unipedicle	LSCS ×2	Pfannenstiel scar	7	None	None
14	50	Invasive breast carcinoma	NSM	Immediate	Bipedicle	LSCS ×2	Pfannenstiel	11	Partial NAC necrosis	None
15	27	Invasive breast carcinoma	MRM	Immediate	Unipedicle	Open appendicectomy	Right paramedian scar	6	None	None
16	56	Bilateral breast carcinoma	Bilateral SSM	Immediate	Bilateral unipedicle	LSCS ×2 + Drain site scar	Pfannenstiel + drain scar	16	None	Superficial umbilical necrosis
17	40	Invasive breast carcinoma	MRM	Immediate	Unipedicle	Open cholecystectomy	Subcostal scar	9	None	Abdominal wound dehiscence
18	44	Invasive breast carcinoma	MRM	Immediate	Unipedicle	LSCS ×1 + Lap appendicectomy	Pfannenstiel + periumbilical	10	None	None
19	29	Malignant phyllodes tumor	Simple mastectomy	Immediate	Unipedicle	LSCS ×2	Pfannenstiel scar	7	None	None
20	52	Invasive breast carcinoma	SSM	Immediate	Bipedicle	LSCS ×2 + open cholecystectomy	Pfannenstiel + subcostal	12	None	Superficial umbilical necrosis

Recipient-site complications were uncommon, with only two patients (10%) developing partial nipple-areola complex necrosis. Donor-site complications occurred in four patients (20%) and included abdominal wound dehiscence (n = 3, 15%) and superficial epidermal necrosis in the umbilical region (n = 2, 10%). No cases of total flap loss were recorded, and the overall flap survival rate was 100%.

No case required abandonment of DIEAP flap reconstruction or conversion to a muscle-sparing transverse rectus abdominis muscle (TRAM) flap. Even in the presence of dense fibrosis or complex scar patterns, successful reconstruction was achieved through intraoperative adaptations such as flap redesign, contralateral hemiabdominal harvest, alternative perforator selection, or venous supercharging. Patient-reported outcomes using the BREAST-Q reconstruction module (Table 2) revealed high levels of satisfaction.

**Table 2:** Postoperative BREAST-Q reconstruction module assessment scores

BREAST-Q parameter	Mean (SD)
Expectation	82.5 (SD: 4.3)
Psychosocial well-being	81.5 (SD: 6.3)
Physical well-being	76.8 (SD: 6.9)
Sexual well-being	74.1 (SD: 15.8)
Satisfaction with breasts	62.3 (SD: 11.0)
Satisfaction with the abdomen	64.8 (SD: 18.3)
Satisfaction with the surgeon	90.5 (SD: 18.9)
Satisfaction with the medical team	94.0 (SD: 9.7)
Satisfaction with quality of life	72.8 (SD: 13.3)

## Discussion

The DIEAP flap breast reconstruction has become the preferred modality for autologous reconstruction due to its advantages in preserving abdominal muscle integrity and minimising donor site morbidity [1]. However, prior abdominal surgeries and resultant scarring have long been perceived as potential impediments to successful flap harvest due to distortion of normal anatomy, fibrosis, and unpredictable perfusion patterns [2,3]. This study uniquely explores the intraoperative realities and decisions required when planning and executing DIEAP flap harvests in scarred abdomens, providing real-world insights that extend beyond standard preoperative imaging interpretations.

While prior literature acknowledges that abdominal scars may alter perforator distribution and vascular architecture, their impact extends beyond imaging findings. These scars often lead to dense subcutaneous fibrosis, altered fascial planes, and challenging intraoperative dissection, factors that can complicate the flap harvest, increase operative time, and predispose to perfusion-related complications [4-7]. However, such technical nuances are seldom elaborated upon in existing studies.

In our case series of 20 patients undergoing DIEAP flap breast reconstruction with prior abdominal scars, the presence of multiple and complex scars was also considered. Scars included Pfannenstiel incisions, subcostal cholecystectomy scars, laparoscopic port site periumbilical scars, and drain site scars. Each presented unique challenges depending on their depth, chronicity, and proximity to major perforators. These scars were not merely surface interruptions; they led to significant underlying fibrosis, distorted fascial planes, and complicated intramuscular dissection of perforators.

Operative planning and intraoperative challenges

**Figure 1:**
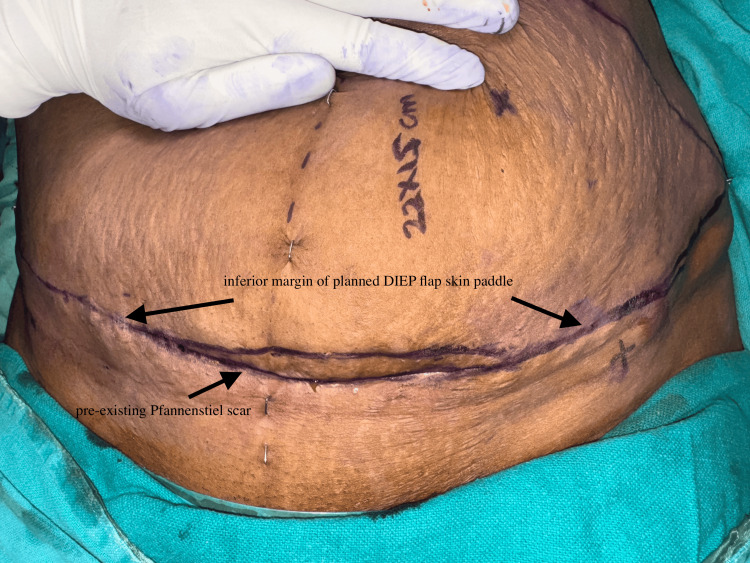
Preoperative marking showing incorporation of a pre-existing Pfannenstiel scar along the inferior margin of the planned DIEAP flap skin paddle. DIEAP: Deep inferior epigastric artery perforator

**Figure 2:**
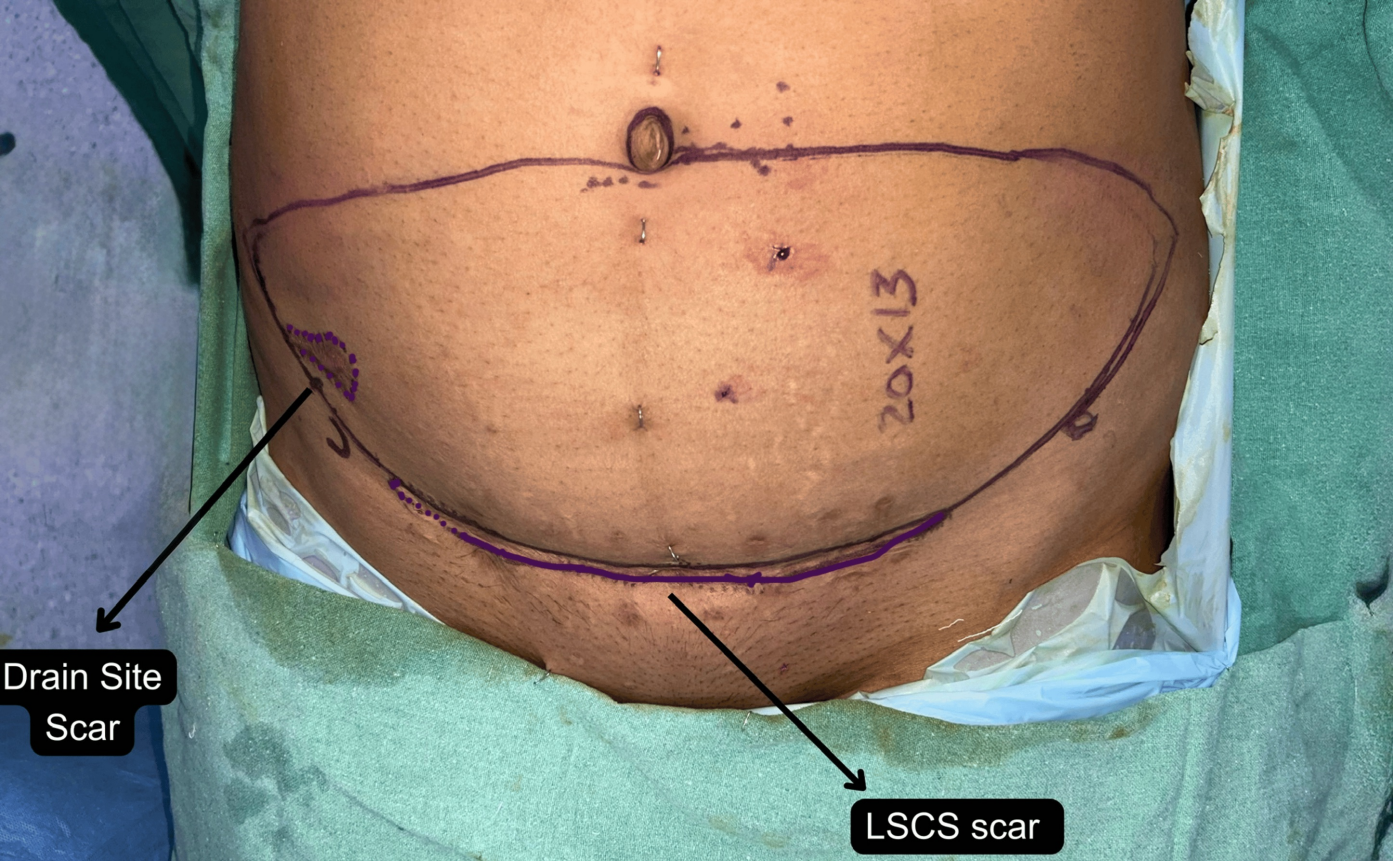
Flap marking in a patient with a prior Pfannenstiel (LSCS) scar and a right-sided drain site scar LSCS: Lower-segment caesarean section

In patients with a Pfannenstiel scar, the abdominal flap was planned to incorporate the low transverse scar along the inferior margin of the skin paddle (Figure 1). The lower abdominal incision is already fixed along the inferior margin of the skin paddle, limiting the freedom to adjust the inferior border of the flap. The superior incision must be determined intraoperatively after assessing whether primary donor site closure can be achieved without tension. Consequently, there is little scope for altering flap design compared to an unscarred abdomen. This restriction is even more pronounced in patients with an additional scar, such as a drain site scar from prior pelvic surgery, where the liberty to modify flap design is significantly decreased, as seen in a patient in our series (Figure 2).

**Figure 3:**
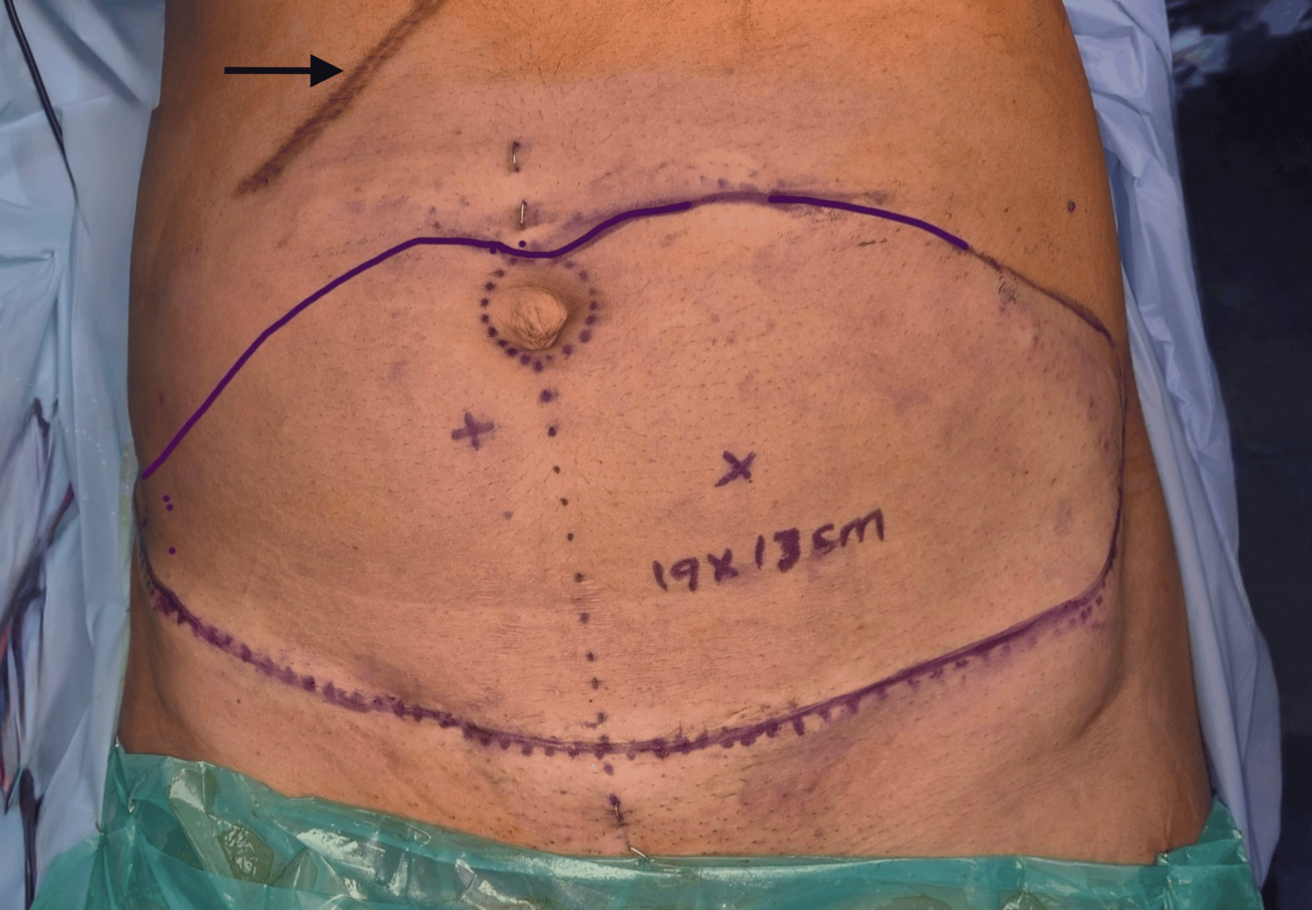
Skewed flap design of DIEAP flap in patients with subcostal scar (black arrow) DIEAP: Deep inferior epigastric artery perforator

In those with a subcostal scar from open cholecystectomy, a flap from the contralateral side was utilised, and an oblique or a skewed design was used (Figure 3). Minimal undermining over the area of the subcostal scar was done during closure for better perfusion of the cranial abdominal flap and to reduce donor site morbidity.

**Figure 4:**
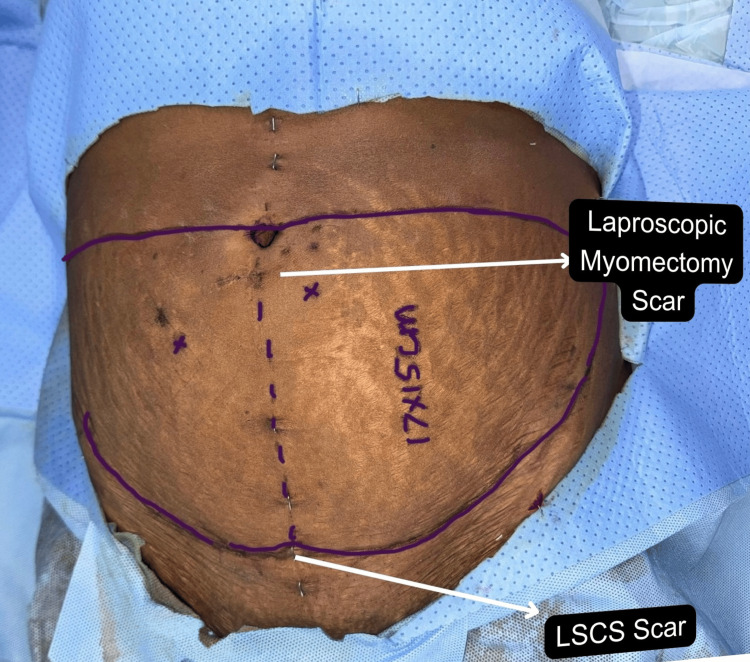
Index patient with a Pfannenstiel scar from a prior LSCS and periumbilical scarring from laparoscopic myomectomy LSCS: Lower-segment caesarean section

For patients with a vertical lower abdomen paramedian scar, the flap from the contralateral side was used, and the flap on the side of the scar was discarded due to unpredictable perfusion patterns and also reduced crossover of perfusion. Although our study showed no significant complications at the donor site in patients with subcostal scars, studies have reported higher rates of donor site complications [8-10]. Among the most illustrative cases was our index patient who presented with bilateral breast carcinoma and a complex abdominal scar profile, i.e., a Pfannenstiel scar from a prior LSCS and periumbilical scarring from a laparoscopic myomectomy (Figure 4).

**Figure 5:**
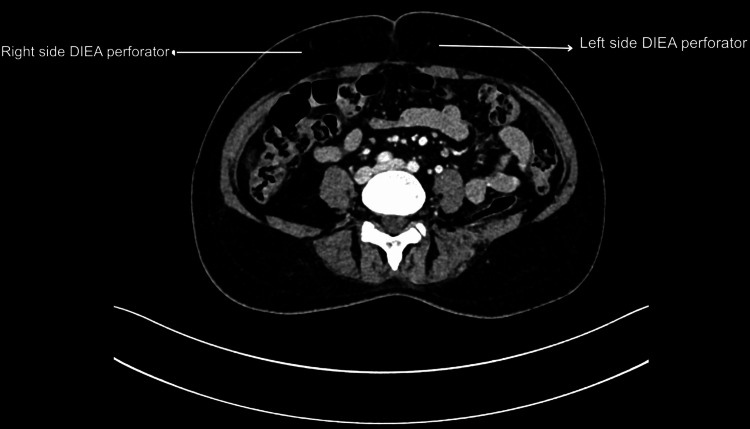
Smaller diameter of deep inferior epigastric perforators bilaterally on CT Imaging DIEA: Deep inferior epigastric artery

**Figure 6:**
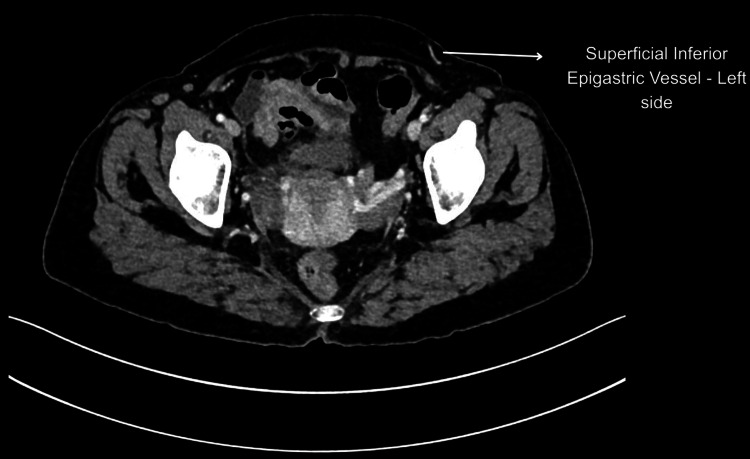
Large calibre of the superficial inferior epigastric vein on CT imaging

Perforator dissection was technically challenging due to dense scar tissue, and intraoperatively, the flap exhibited a dark, congested appearance when only the deep system was anastomosed. This observation indicated compromised venous outflow via the deep system, necessitating venous supercharging through anastomosis of the superficial inferior epigastric vein (SIEV) to the cephalic vein. Previous studies have shown that in patients with Pfannesteil incisions, the perforator size was larger and the flap was perfused better due to ischaemic preconditions in comparison to the no-abdominal-scar group [11-13]. Laparoscopic port sites were found to have no influence on the perfusion pattern. On the contrary, in our study, this patient had narrow DIEA perforators and a larger superficial epigastric venous system bilaterally on preoperative imaging (Figures 5-6) as well as intraoperatively.

**Figure 7:**
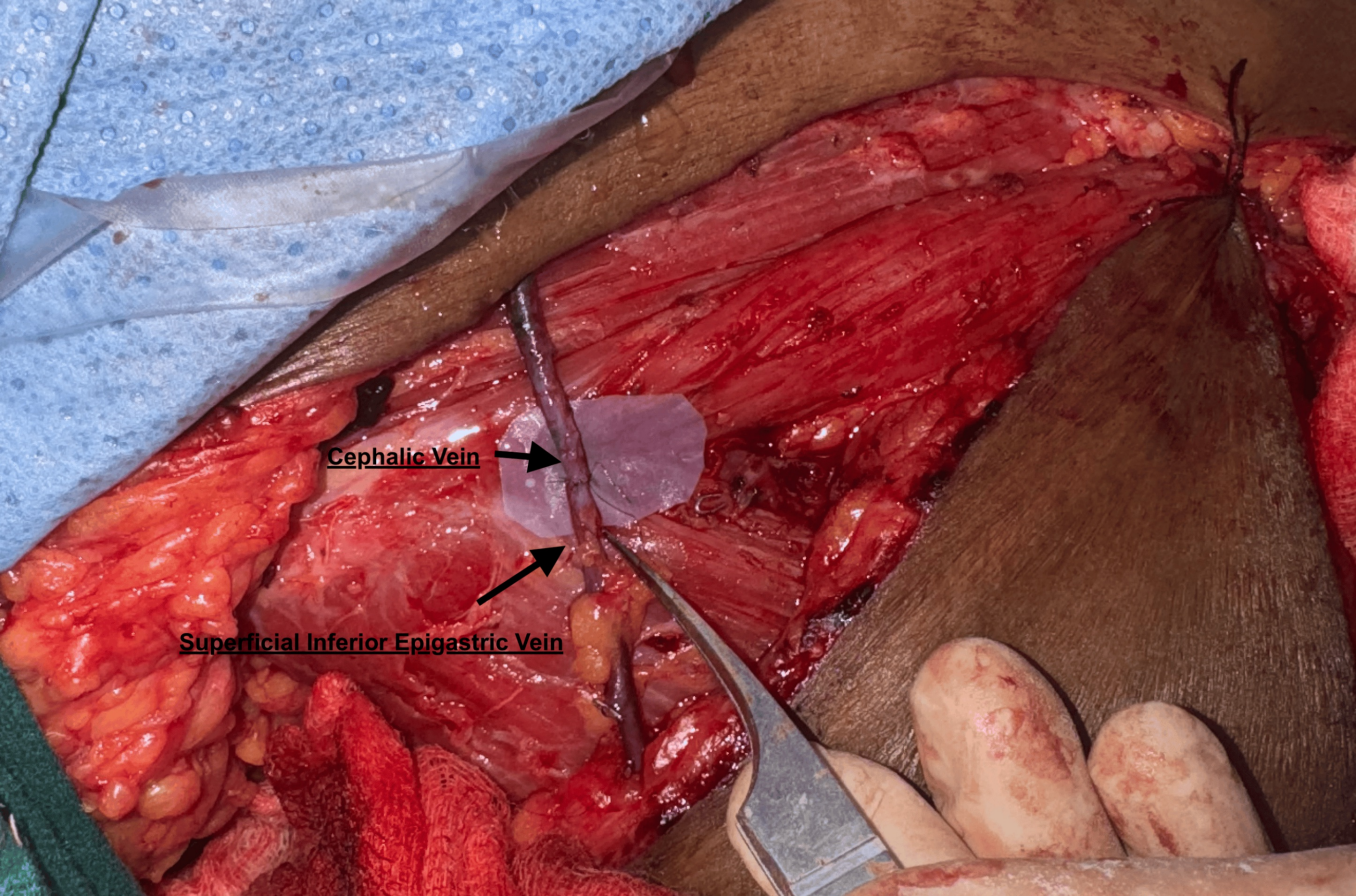
Venous supercharging using the superficial epigastric vein anastomosed to the cephalic vein

**Figure 8:**
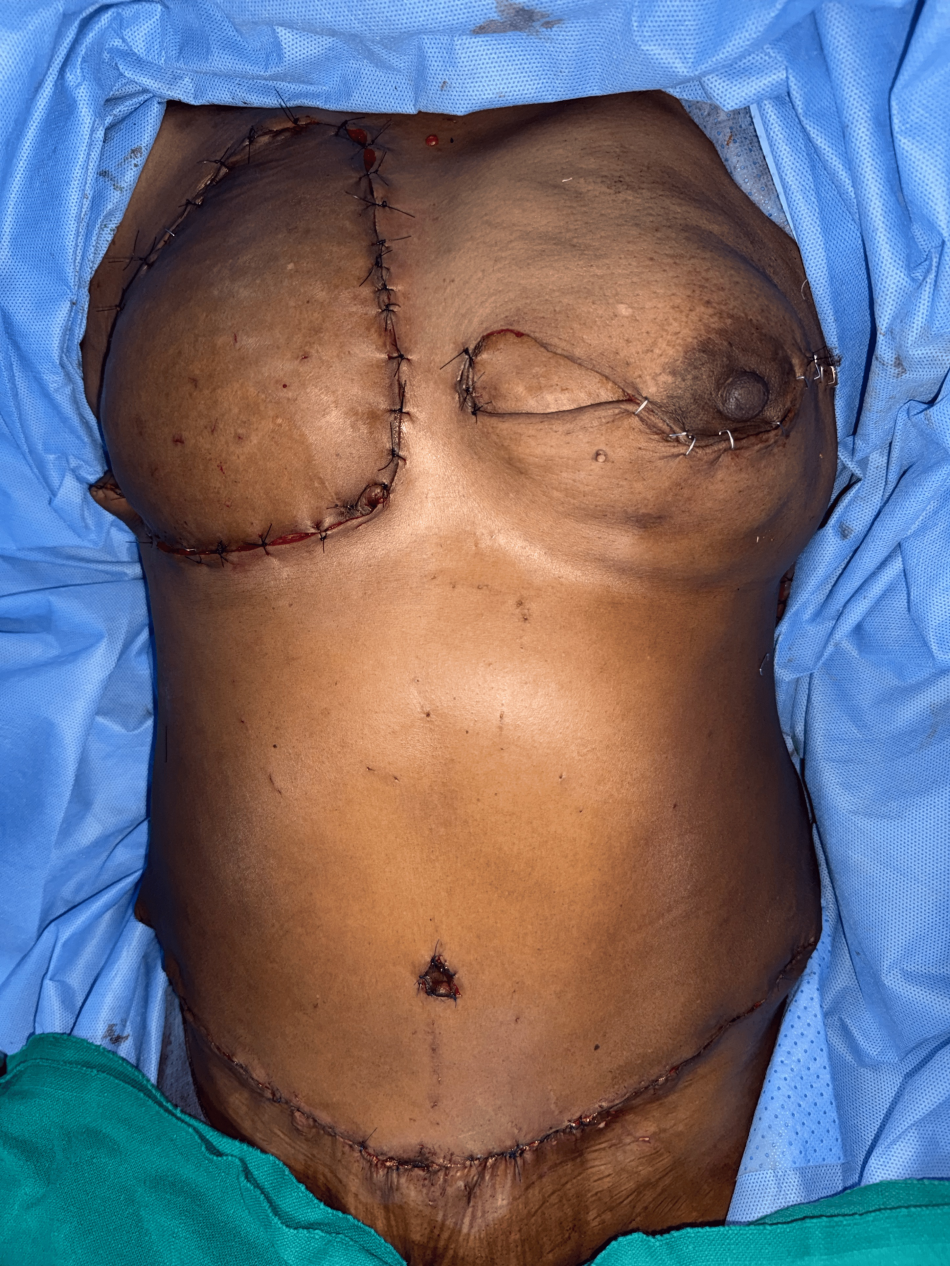
Imediate postoperative picture of the index patient

Upon performing a venous supercharging using the superficial epigastric vein anastomosed to the cephalic vein, the flap rapidly improved, with bright red bleeding returning, and ultimately survived well (Figures 7-8). This case underlines the critical role of intraoperative judgement and adaptability in salvaging flaps, particularly when dominant superficial systems are encountered, an observation supported in part by Varnaava et al., who noted the occasional necessity of venous augmentation in high-risk cases [14].

**Figure 9:**
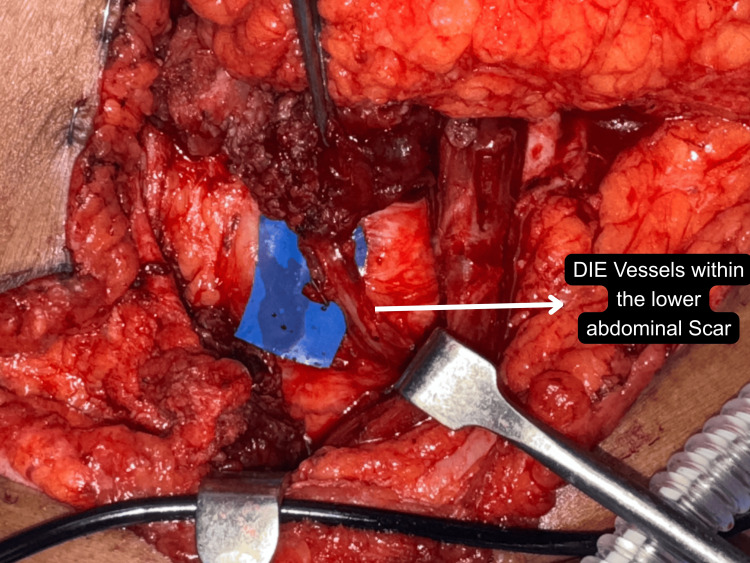
Challenges in dissection of the perforator within the scar intraoperatively DIE: Deep inferior epigastric

**Figure 10:**
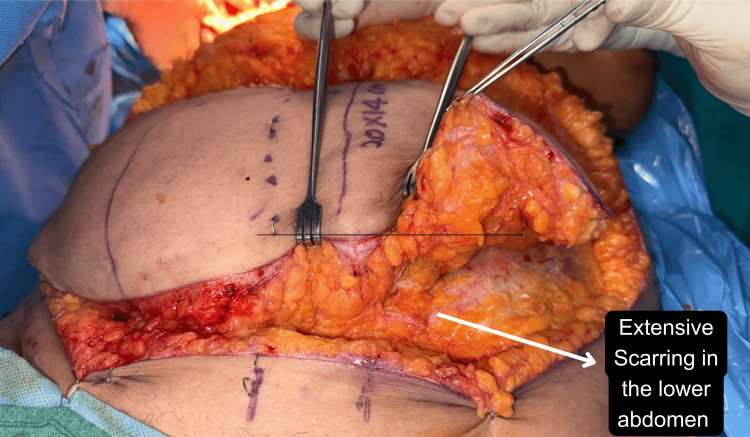
Scarring in the lower abdomen in a patient with a previous LSCS scar LSCS: Lower-segment caesarean section

Fibrotic tissue planes not only lengthened operative time but also increased the difficulty of perforator dissection (Figures 9-10). Despite these intraoperative difficulties, all flaps survived without total loss, and only minor revisions were required, confirming that prior abdominal surgery is not a contraindication to DIEAP reconstruction but rather a factor that demands individualised planning and expert execution. The use of CTA significantly aided in visualising the extent of the scar and mapping reliable perforators even in scarred zones.

In terms of postoperative outcomes, patients in our series reported high satisfaction across BREAST-Q domains, especially with psychosocial and surgeon-related domains. Interestingly, even the index patient with complex scarring and intraoperative complications reported satisfactory aesthetic and functional outcomes, reinforcing that good results are achievable even in complex cases, provided the challenges are recognised and addressed proactively. All cases of abdominal wound dehiscence occurred in patients with multiple scars or those with obesity and high BMI, suggesting that these factors increase the cumulative scar burden and compromise tissue quality, thus leading to high donor-site morbidity. While the sample size precludes definitive risk stratification, heightened vigilance and tension-minimising closure strategies are recommended in such patients. When compared to the established normative data [15], our BREAST-Q scores revealed a notably higher level of satisfaction with the breast: approximately 10% greater (62.3 compared to 58). Scores for satisfaction with the abdomen were slightly lower (64.8 versus 78); psychosocial well-being was better than normative values (81.5 versus 71). Scores for physical well-being were found to be slightly lower than the reference values (76.8 versus 83). We acknowledge and commend Payne and colleagues for their significant and practical contribution to establishing normative values for each BREAST-Q module [16]. Our results are also consistent with the study conducted by Razzano et al. [17].

This study is limited by its small sample size, single-centre design, and descriptive methodology, which restricts statistical inference and generalisability. Nevertheless, its prospective nature provides valuable qualitative insight into intraoperative challenges encountered during DIEAP flap harvest in scarred abdomens. Operative variables were not formally quantified, reflecting the exploratory intent of documenting adaptive surgical strategies rather than establishing causal relationships. Future multicentre studies incorporating standardised intraoperative criteria, preoperative high-resolution ultrasonography with colour Doppler in a scarred abdomen, quantitative perfusion analysis assessments (e.g., indocyanine green angiography), and advanced vascular modelling may help better delineate the impact of specific scar patterns on perforator integrity and venous drainage.

## Conclusions

This study provides meaningful insight into the technical nuances and intraoperative decision-making involved in DIEAP flap harvest from scarred abdomens. Successful reconstruction in these challenging cases depends on meticulous preoperative planning, careful perforator mapping, and the ability to modify intraoperative strategies in response to altered vascular anatomy. Abdominal scars do not contraindicate DIEAP flap breast reconstruction, but demand heightened surgical vigilance and flexibility. A thorough understanding of scar-induced anatomical variations, coupled with judicious use of preoperative imaging and intraoperative judgement, can ensure optimal flap viability, aesthetic outcomes, and patient satisfaction even in complex scarred abdomens.
